# The Hip Morphology Changes with Ageing in Asian Population

**DOI:** 10.1155/2018/1507979

**Published:** 2018-09-27

**Authors:** Yingchao Yin, Ruipeng Zhang, Lin Jin, Shilun Li, Zhiyong Hou, Yingze Zhang

**Affiliations:** ^1^Department of Orthopaedic Surgery, The Third Hospital of Hebei Medical University, Shijiazhuang, Hebei 050051, China; ^2^Key Laboratory of Biomechanics of Hebei Province, Shijiazhuang, Hebei 050051, China

## Abstract

**Objectives:**

This study aims to determine the changing in hip anatomy parameters with age and reveals the reason for the extorsion of lower extremity in the aged.

**Design:**

Retrospective study.

**Participants:**

One hundred and forty patients who had received imaging check of the femur and acetabulum between October 2013 and October 2016 were included in this study.

**Main Outcome Measures:**

The femoral neck torsion angle (FNTA), neck-shaft angle (NSA), and acetabular anteversion angle (AVA) were measured by an experienced orthopedic surgeon. All the patients' demographic and physical characteristics including age, sex, body laterality, height, and weight were recorded. The Student* t*-test, two-way ANOVA, Pearson correlation, and multiple linear regression were used for the statistical analysis.

**Results:**

The mean age for male and female was 45.01±15.38 and 49.30±17.63 years, respectively. Outcomes revealed that the NSA on the right side of the body, 133.46±4.46° in male and 134.36±4.71° in female, was statistically higher than the left side. Female FNTA had significantly higher values than male (*P*<0.01). Two-way ANOVA reveals that FNTA and AVA were correlated with age (P<0.05) but not weight, height, or BMI. NSA was correlated with age, weight, and BMI (P<0.05) but not height. Multiple linear regression analysis showed that only age made an independent contribution to NSA.

**Conclusions:**

The NSA and FNTA of Asian population may have an obvious decrease whereas AVA increases with ageing, which reveals the reason for the extorsion of lower extremity with elderly. During hip-related surgery in elderly patients, more attention should be paid to these lower extremity anatomic changes.

## 1. Introduction

As the population ages, orthopedists will increasingly face age-related hip diseases. Old people have changed a lot of their physical posture compared to young people, such as kyphosis of the dorsal spine, stature shrinking, and extortion of the lower extremities. Vertebral wedge deformity caused by senile osteoporosis is the main reason for the height loss [1, 2]. However, few studies were done to explore the morphological change of the lower extremity in the aged. Are all of these changes in the gesture due to the changes in skeleton or soft tissue balance?

Neck-shaft angle (NSA), femoral neck torsion angle (FNTA), and acetabular anteversion angle (AVA) are three important hip morphology parameters. They varied according to several previous studies. They might be affected by physical characteristics, such as age, sex, body laterality, and ethnicity [3, 4]. External factors, such as climate, clothing, and lifestyle, could also be important factors to affect these parameters [5]. Due to the position attained by the foetus in utero, a high value of FNTA is normally seen in the femoral neck [6]. The FNTA is about 30 to 40 degrees at birth, and the value gradual decreased to 10-15 degrees by early adolescence [7]. Although several similar studies were conducted, the analysis is insufficient regarding the effects of age, height, and weight on the hip morphology parameters.

Hip morphology parameters, such as NSA and FNTA, are essential for the diagnosis and treatment of pathological hip diseases including developmental dysplasia of the hip (DDH) [8], Perthes disease [9], and proximal femoral fractures [10]. Recent researchers have reported that abnormal FNTA or AVA participate in the pathogenesis of hip osteoarthritis [11], gluteal tendinopathy [12], femoroacetabular impingement (FAI), and cerebral palsy [13, 14]. Additionally, an incompatibility prosthetic during total hip arthroplasty could result in limited range of motion, dislocation, and aseptic dislocation [15, 16]. To avoid these complications, FNTA and AVA should be given full preoperative and intraoperative consideration. A good understanding of the hip morphology trend could increase the accuracy of hip surgery, especially for the patients with abnormal anatomy.

The purpose of the present study was to explore the changes of hip morphology parameters with ageing in Asian adults. We hypothesized that these three hip morphology parameters might be affected with ageing by demographic and physical properties.

## 2. Materials and Methods

### 2.1. Patients

This study was designed as a retrospective study and aimed to explore the influences of demographic and physical properties on hip morphology. A total of 140 Asian adults (70 males and 70 females) who received CT scan of the hip and femur between October 2013 and October 2016 were included. The mean age for male and female were 45.01±15.38 years (range, 18-83 years) and 49.30±17.63 years (range, 18-88 years). The male and female were 71.65±12.07 and 59.96±9.98 kg weight, respectively, with an average height of 1.73±0.06 and 1.63±0.07 m. Written consent from all the included participants was waived due to the retrospective design of this study. However, the patient's information was anonymized during this study. The measurement of hip morphology was conducted using picture archiving and communication systems (PACS). Inclusion criteria for the study were Asian patients: (1) with eligible imaging data for measurement, (2) without previous hip disorders that might influence hip morphology. All the demographic data of each participant were recorded, including age, weight, and height. NSA, FNTA, and AVA were measured in all the subjects.

### 2.2. Measurement Methods

The femoral neck oblique axial CT reconstruction method was used to measure FNTA [17], which provides more accurate anteversion assessment than axial images. There are two steps to measure the FNTA. First, reconstruct the femoral neck along its axis at the CT workstation. Then, subtract the femur condylar angle. The axis of the femoral neck was defined as the line connecting the center of the femoral head, and the center of the femoral neck. Neck-shaft angle, measured in standard anterior-posterior X-rays of the pelvis, was the intersection angle between the femoral neck axis and proximal femoral shaft axis. Acetabular anteversion, defined as the angle formed by a line connected the anterior and posterior acetabular ridge and another line perpendicular to the coronal plane of the body, was measured on axial CT images through the acetabular center [4].

### 2.3. Statistical Analysis

Statistical analysis was performed by IBM SPSS Statistics for Windows, Version 21.0 (IBM Corp., Armonk, NY, USA). Continuous data were presented as the mean and standard deviation. The distributions of all variables were evaluated for normality using a combination of quantile-quantile (Q-Q) plots and Shapiro-Wilk tests. The sex and side differences in each hip morphology parameter were compared using two-way ANOVA. Pearson correlation coefficient analysis was used to analyze potential relationship between demographic data and hip parameters. Correlation results were interpreted as no or very week (-0.1 to 0.1), weak (-0.3 to -0.1 or 0.1 to 0.3), moderate (-0.5 to -0.3 or 0.3 to 0.5), and strong (-1.0 to -0.5 or 1.0 to 0.5) [18]. Multiple linear regression was conducted to assess the independent association between demographic properties and NSA.* P*<0.05 (two-tailed) was considered statistically significant. A highly significant difference was defined as* P*<0.01 (two-tailed).

## 3. Results

### 3.1. Demographic Data

The demographic data of all the participants were showed in the Table 1. The body mass index (BMI) is equal to the weight divided by the square of height. The mean BMI of male and female were 23.96±4.21 and 22.59±3.93 kg/m^2^ (*P*=0.05) (Table 1).

### 3.2. Laterality-Sex-Based Analysis

NSA, FNTA, and AVA, all these three hip morphology parameters measured for both sides in all the subjects, were analyzed with two-way ANOVA and shown in Table 2. NSA on the right side of the body was statistically higher than the left side (*P*<0.01). Female had a significantly larger FNTA compared with male (*P*<0.01) (Table 2).

### 3.3. Pearson Correlation Analysis with Age, Weight, Height, BMI, and Hip Morphology Parameters

Outcomes of Pearson correlation test showed that FNTA was correlated with age (P<0.05) but not weight, height, or BMI except height on the right side in the male. AVA was correlated with age (P<0.05) but not weight, height, or BMI. However, NSA was correlated with age, weight, and BMI (P<0.05) but not height. All the Pearson correlation coefficients (*r* value) were showed in the Tables 3 and 4.

### 3.4. Multiple Linear Regression for the Relationship between NSA and Age and Weight and BMI

Multiple linear regression analysis was performed using NSA as dependent variable and age, weight, and BMI as independent variables. The results demonstrated that only age made an independent contribution to NSA in both side and sex (Table 5).

## 4. Discussion

NSA, FNTA, and AVA are three important hip morphology parameters. A good understanding of the normal range of them is beneficial for the reduction of the fracture fragments and prosthesis placement. Some pathogenic mechanisms of hip diseases are related to the above three parameters, such as hip osteoarthritis, developmental dysplasia of the hip (DDH), femoroacetabular impingement (FAI), and cerebral palsy (CP). Therefore, exploring the variation tendency of hip morphology parameters in Asian population is beneficial to the diagnosis and treatment of hip-related diseases.

**Figure 1:**
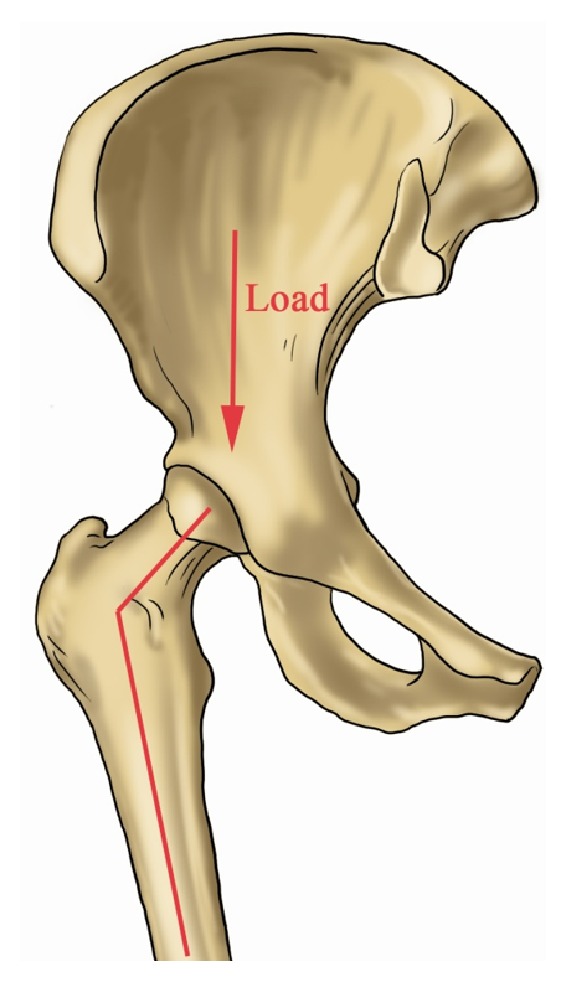
The illustration shows the weight of the upper part of body transmitted from the hip to the lower limbs.

We found that NSA in Asian adults has a significant negative correlation with age, weight, and BMI. However, the multiple linear regression analysis revealed that only age made an independent contribution to NSA. Geoffrey [19] used finite element models to analyze what the effect of NSA on hip stress. Their study outcomes revealed that a varus NSA is subjected to higher mechanical stress than those with a normal femoral neck angle. Ruff et al. conducted another study to investigate the relationship between mechanical loading (body weight) and proximal femoral dimensions [20]. They found that the proximal femoral diaphyseal size is more closely correlated to the current body weight than that at initial skeletal maturity (18 years). As we know, the weight of upper limbs and trunk was transmitted from the hip to the lower limbs (Figure 1). With the increase of age, the proximal of femur was suffered the repeated impact from the acetabulum. As all the researchers reported, the body weight may be one of the reasons to explain the NSA decreasing along with age. Gilligan [5] assembled a global NSA database comprising over 8000 femora representing 100 human group. The results showed that there is no sex difference, but possibly a small lateral difference. They think the lateral difference could be due to right leg dominance. A total of 140 subjects were included in this study, and the outcome of statistical analysis shows the right side of body has a larger NSA than the left. We thought this phenomenon might also be affected by the habitual dominance of lower limb.

FNTA is the angle between the femoral neck axis and coronal plane of the femoral condyles. As one of the important parameter of hip morphology and lower extremity alignment, FNTA plays an important role in lower extremity function [21]. Kingsley et al. [6] measured 630 American adults' femurs. The outcome confirmed that infants have a high degree of FNTA, and it gradually recedes to that seen in the adult. But, the difference between the male and female is small. However, Wright et al. [22] indicated that female had a significantly greater FNTA than male (P<0.05) in 60 octogenarian Netherlanders. Race and geography may be factors accounting for the variations of FNTA in addition to demographic properties. Our measurements revealed that female have higher values of FNTA than male. The FNTA and AVA were changed with ageing for all the people.

**Figure 2:**
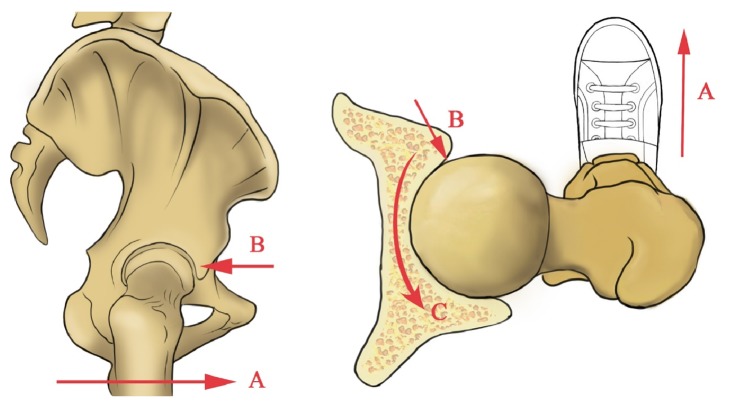
The “A” arrow represents the body which was walking forward. The “B” arrow means that the femoral head was bearing a backward pressure from the anterior acetabular wall to maintain the body moving forward. The “C” arrow represents the changing of acetabular wall as the people ageing.

Bone absorption and regeneration can lead to changes in the skeleton morphology of the hip. Ruff and Hayes investigated 103 femurs and 99 tibias to explore the changes of lower limb with ageing [23]. The results demonstrated that both male and female undergo endosteal resorption and medullary expansion with ageing. The elderly are more likely to undergo the hip fracture by because of senile osteoporosis. Okazaki et al. extracted the subchondral bone columns from the femoral head of 11 female patients with a femoral neck fracture [24]. It is found that microfractures might occur during the daily activities in the development of osteoporosis. As we can see from the Figure 2, the body was walking forward as the “A” arrow pointed. The distal of the femur suffered a forward force. Then the power transmits from the hip through acetabulum to the body, which makes the people moving forward. The proximal of the femur bears a backward force from the anterior acetabular wall during walking. The backward force continually and repeatedly impacts on proximal femur during daily walking, which makes the FNTA inclined and the AVA increased with ageing [25]. Our measurements got the similar results of changes of the FNTA and AVA. These changes also revealed the phenomena of the lower extremity extorsion with elderly.

Our study has several limitations. First, the measurements of FNTA, NSA, and AVA we reported cannot lead to a comprehensive understanding of hip in Asian adult population because there are still many other parameters of hip morphology, such as femoral head diameter, acetabular depth, and acetabular abduction angle. Second, the number of the subjects in this present study was not large enough to represent the Asian adult hip morphology. Moreover, our hypothesis about hip morphology changes was based on the data analysis of this study, and future biomechanics and long-term follow-up studies are needed.

In summary, our study found that the NSA and FNTA of Asian population has an obvious decrease with ageing, which reveals the reason for the extorsion of lower extremity in the aged. In the treatment of elderly patients with hip disease such as hip replacement and internal fixation of intertrochanteric fractures, more attention should be paid to these changes.

## Figures and Tables

**Table 1:** Demographic data of the participants.

	Male	Female	*P*
N	70	70	
Age (y)	45.01±15.38	49.30±17.63	0.13
Weight (kg)	71.65±12.07	59.96±9.98	<0.01
Height (m)	1.73±0.06	1.63±0.07	<0.01
BMI (kg/m^2^)	23.96±4.21	22.59±3.93	0.05

**Table 2:** Differences in hip morphology between sex and sides (*P*1 value for sex;* P*2 value for side; *P*3 value for interaction).

	Male		Female		*P1*	*P2*	*P3*
	Left	Right	Left	Right			
NSA (°)	131.93±5.39	133.46±4.46	132.41±3.95	134.36±4.71	0.22	<0.01	0.70
FNTA (°)	19.68±7.42	20.37±8.51	24.13±9.17	23.50±9.56	<0.01	0.98	0.53
AVA (°)	18.26±4.80	19.05±5.42	18.24±5.79	19.74±6.11	0.61	0.09	0.59

**Table 3:** The correlation (*r* value) between hip parameters and physical properties in the male (^*∗*^*P*<0.05).

	NSA	FNTA	AVA
	*r*	*r*	*r*
**Left**			
Age	-0.335^∗^	-0.259^∗^	0.274^∗^
Weight	-0.316^∗^	-0.105	0.124
Height	0.074	0.097	-0.067
BMI	-0.342^∗^	-0.148	0.160
**Right**			
Age	-0.362^∗^	-0.311^∗^	0.463^∗^
Weight	-0.367^∗^	-0.076	0.010
Height	0.038	0.255^∗^	0.022
BMI	-0.389^∗^	-0.187	0.027

**Table 4:** The correlation (*r* value) between hip parameters and physical properties in the female (^*∗*^*P*<0.05).

	NSA	FNTA	AVA
	*r*	*r*	*r*
**Left**			
Age	-0.282^∗^	-0.269^∗^	0.258^∗^
Weight	-0.282^∗^	-0.053	0.082
Height	-0.041	0.088	-0.160
BMI	-0.255^∗^	-0.099	0.158
**Right**			
Age	-0.270^∗^	-0.309^∗^	0.312^∗^
Weight	-0.358^∗^	-0.085	0.008
Height	0.005	0.214	-0.213
BMI	-0.336^∗^	-0.202	0.107

**Table 5:** Multiple regression analysis for the relationship between NSA as dependent variable and age, weight, and BMI as independent variables (^∗^*P*<0.05).

	Male		Female	
	Dominant side NSA	Non-dominant side NSA	Dominant side NSA	Non-dominant side NSA
Age *β* Coef (SE)	-0.095 (0.041)^∗^	-0.084 (0.033)^∗^	-0.067 (0.026)^∗^	-0.074 (0.031)^∗^
Weight *β* Coef (SE)	-0.043 (0.133)	-0.058 (0.107)	-0.160 (0.096)	-0.196 (0.112)
BMI *β* Coef (SE)	-0.237 (0.387)	-0.181 (0.311)	0.140 (0.246)	0.079 (0.287)

## Data Availability

The data used to support the findings of this study are available from the corresponding author upon request. Additional data from this study are available from the corresponding author.
